# Detection of Apparent Cell-free *M*. *tuberculosis* DNA from Plasma

**DOI:** 10.1038/s41598-017-17683-6

**Published:** 2018-01-12

**Authors:** E. S. Click, W. Murithi, G. S. Ouma, K. McCarthy, M. Willby, S. Musau, H. Alexander, E. Pevzner, J. Posey, K. P. Cain

**Affiliations:** 1 Centers for Disease Control and Prevention, Atlanta, GA USA; 2 Kenya Medical Research Institute, Kisumu, Kenya; 3 Centers for Disease Control and Prevention, Kisumu, Kenya

## Abstract

New diagnostics are needed to improve clinicians’ ability to detect tuberculosis (TB) disease in key populations such as children and persons living with HIV and to rapidly detect drug resistance. Circulating cell-free DNA (ccfDNA) in plasma is a diagnostic target in new obstetric and oncologic applications, but its utility for diagnosing TB is not known. Here we show that *Mycobacterium tuberculosis* complex DNA can be detected in plasma of persons with sputum smear-positive TB, even in the absence of mycobacteremia. Among 40 participants with bacteriologically-confirmed smear-positive TB disease who had plasma tested by quantitative PCR (qPCR), 18/40 (45%) had a positive result on at least one triplicate reaction. Our results suggest that plasma DNA may be a useful target for improving clinicians’ ability to diagnose TB. We anticipate these findings to be the starting point for optimized methods of TB ccfDNA testing and sequence-based diagnostic applications such as molecular detection of drug resistance.

## Introduction

Tuberculosis (TB) is the leading infectious cause of death globally, though it is preventable, treatable and curable. In 2015 there were an estimated 10.4 million new cases worldwide, including at least 1 million cases in children and 480,000 new cases of multi-drug resistant TB (resistant to at least rifampin and isoniazid)^1^. Globally, however, one third of cases go undetected and unreported, presumably due in part to inadequate diagnostics. Mycobacterial culture is the reference standard for TB diagnosis but is not widely available in resource-limited settings. Sputum microscopy, pioneered by Robert Koch in 1882, remains the mainstay of diagnosis globally. Drug susceptibility testing is critical to ensuring people with TB are on appropriate treatment but is also not widely available in resource-limited settings. The cartridge-based Xpert MTB/RIF® (“Xpert”) real-time PCR assay for molecular detection of *Mycobacterium tuberculosis complex* (MTBC) and rifampin resistance has improved sensitivity over microscopy and allows for rapid detection of resistance to a key drug in the treatment of TB^2,3^. However, important diagnostic gaps remain. Therefore, there is an urgent need for improved diagnostic approaches, both for detection of disease and for detection of drug resistance in order to guide appropriate treatment for persons with TB.

Most existing tests for detecting TB and identifying drug resistance require a sputum sample. Even when the gold standard culture is available, diagnostic yield from sputum is poor in patient populations such as young children, persons with extrapulmonary TB, and persons living with HIV. Further, bacteria that grow in culture may not be fully representative of the bacterial populations within a person with active disease^4^. Drug resistance testing (DST) generally relies on a culture isolate. Given the slow growth of MTBC this can delay results for months and lead to ineffective treatment regimens and acquisition of resistance to additional drugs. Blood would be an attractive sample type, particularly in persons without pulmonary TB or who are not able to produce sputum.

Human circulating cell-free DNA (ccfDNA) results from cellular turnover and release of small fragments of DNA into the bloodstream. There are examples of the diagnostic utility of detecting human DNA fragments in such applications as early prenatal fetal genetic testing, where fragments of fetal DNA are detected in maternal plasma^5^. Detection of cell-free DNA in plasma is also being explored as a method of “liquid biopsy” for cancers^6^. We sought to determine whether DNA from *M*. *tuberculosis* can be detected in the plasma of persons with TB disease. If so, it could be a possible target for improving clinicians’ ability to detect TB and to obtain molecular data in a more timely fashion than is currently available.

## Methods

### Study participants

We recruited adults ≥18 years from clinics and hospitals in Kisumu County, Kenya with TB diagnosed by positive sputum smear microscopy and on treatment ≤7 days.

### Specimen collection procedures

Sputum and urine were collected in 50 mL falcon sterile containers (Beckton Dickinson (BD), Franklin Lakes, NJ). Blood (5 mL) was collected directly into Myco/F Lytic bottles (Becton Dickinson, Franklin Lakes, NJ) for mycobacterial culture, and 10 mL was collected directly into K_2_EDTA Vacutainer tubes (BD) for plasma preparation.

HIV testing was offered to all participants with unknown status or with negative results from testing performed more than three months prior to enrollment.

Urine (approximately 50 mL) was centrifuged at 3,000 x g for 10 minutes and then the supernatant was decanted to leave approximately 5 mL specimen. Sputum and concentrated urine were then processed using the standard N-acetyl-L-cysteine/4% sodium hydroxide-2.9% sodium citrate (NALC/NaOH Na-citrate, final concentration of NaOH 1%) method^7^. Following centrifugation, specimens were decanted and resuspended in 2.0 mL of fresh phosphate buffer (pH 6.8). The concentrated pellet was mixed completely and a total of 0.5 mL of processed specimen was inoculated into one Mycobacterial Growth Indicator Tube [(MGIT), BD]. Myco-F/Lytic blood cultures were placed directly into the BD BACTEC^TM^ 9120 instrument.

Plasma was prepared by centrifugation of blood EDTA tubes at 1,500 x g for 10 minutes (“single-spin” plasma). Plasma to be used for DNA preparation (“double-spin” plasma) was transferred to four microfuge tubes and centrifuged at 16,000 x g for 10 minutes. Supernatants were carefully removed from each tube, pooled, and stored at −80 °C. Target time from blood collection to plasma preparation was 2 hours, but samples were accepted at any time within the same day for processing.

DNA was prepared from 4 mL of plasma using the QiaAmp Circulating Nucleic Acid Kit (Qiagen, Hilden, Germany) and eluted with 20 μL buffer AVE.

HIV testing was performed according to the Kenya national guidelines using two separate rapid serologic tests. If the first rapid test was positive, a second rapid test was performed for confirmation.

### Culture

MGIT cultures were incubated in the MGIT 960^TM^ instrument and Myco/F lytic bottles were incubated in the BD BACTEC^TM^ 9120 instrument for up to 6 weeks each. Cultures flagged as positive were inoculated to brain heart infusion media to determine the presence of contamination by non-mycobacterial species; smears of culture broth were also prepared and stained using Ziehl-Neelsen stain and bright-field microscopy to confirm the presence of acid-fast bacilli (AFB). BACTEC cultures that were positive by AFB microscopy were sub-cultured to Lowenstein Jensen media and incubated at 37 °C. MGIT and BACTEC cultures demonstrating growth consistent with *Mycobacterium* sp. were identified using the MGIT TBc Identification Test (Beckton Dickinson Diagnostics, Spark, MD) and Capilia TB Neo (Tauns, Kozuwa, Japan), respectively. MGIT cultures with both *Mycobacterium* sp. and contaminating species were re-decontaminated using the standard method described above.

### Xpert testing

Xpert testing (Cepheid, Sunnyvale, CA) of sputum was performed by mixing 0.75 mL of the processed sputum pellet with sample buffer at a ratio of 2:1 and processed and tested according to the manufacturer’s instructions. We performed Xpert testing on single-spin plasma according to the manufacturer’s protocol for testing of sputum. Xpert testing of plasma was discontinued after approximately 30 tests were performed with no positive results.

### Real-time PCR

Real-time PCR (qPCR) was performed using primers (F: 5′-CCTACTACGACCACATCA-3′; R: 5′-CCGTAAACACCGTAGTTG-3′; probe: 5′-/56-FAM/ATGTGCTCCTTGAGTTCGCCAT/3BHQ_1/-3′, Integrated DNA Technologies, Coralville, Iowa) that amplify a 106 base pair region of IS6110 in *M*. *tuberculosis* and were found to not amplify a product when tested against a panel of non-tuberculous mycobacteria and other respiratory pathogens (M. Willby, personal communication). Reaction mixture (20 μL total) included 1X Lightcycler Mix (Roche, Basel, Switzerland) 2 or 3 μL DNA template, and 300nM final concentration each of forward and reverse primers and probe (Integrated DNA Technologies, Coralville, Iowa). Cycling and detection was performed on the Applied Biosystems 7500 Fast Real-Time PCR System using the following conditions: initial incubation at 95 °C for five minutes, 45 cycles of 95 °C for 15 seconds and 60 °C for one minute. Samples were tested in triplicate. A qPCR reaction targeting human RNAseP was used to confirm that DNA had been purified from each sample tested^8^.

### Ethical approval

This study was reviewed and approved by the CDC and Kenya Medical Research Institute IRBs (CDC Protocol #6484) and all experiments were performed in accordance with relevant guidelines and regulations. All participants provided written informed consent.

### Data availability

The datasets generated during and/or analysed during the current study are available from the corresponding author on reasonable request.

## Results

A total of 55 potential participants were screened, 51 were determined to be eligible, and of these one declined enrollment. A total of 50 participants were enrolled, including 32 (64%) with HIV and 18 (36%) without HIV infection; all but 3 participants consented to having blood drawn (Table 1). Among participants with HIV, median CD4 was 238 cells/mm^3^ (interquartile range 96–340). All participants were enrolled on the basis of having a positive sputum smear result as performed under routine program conditions. Of the 50 participants, 47 (94%) participants had sputum, blood, or urine positive for MTBC by Xpert and/or liquid culture (bacteriologically confirmed TB disease); all participants with blood or urine culture positive for MTBC also had a positive sputum Xpert and/or culture. Of the three participants who did not have confirmation of sputum positive for MTBC, one had sputum tested by both Xpert and culture and two had sputum tested by Xpert only.

**Table 1:** Characteristics of Study Participants.

Characteristic	Total (n = 50)
	n (%)
Median age at enrollment (years, interquartile range)	32 (28–37)
Male sex – No. (%)	28 (56)
HIV	
Positive	32 (64)
Negative	18 (36)
CD4 count (cells/mm^3^)(interquartile range)	238 (96–340)

Of 43 plasma DNA samples tested by qPCR, 19 (44%) were positive in at least one of the triplicate reactions (Table 2). All no-DNA template control reactions were negative. Among 40 participants with bacteriologically-confirmed TB disease who had plasma tested by qPCR, 18/40 (45%) were positive in at least one triplicate reaction; of these, one was positive by culture of sputum but not by Xpert testing of sputum. One specimen was positive by qPCR but negative by Xpert testing of sputum and by culture of blood and urine; culture of sputum was not done. Of three participants with mycobacteremia, two were positive by qPCR. Cycle threshold (Ct) scores for all positive IS6110 qPCR reactions were above 33 (median 38, IQR 37–39). A comparison of individual and mean sputum Xpert Ct scores and plasma qPCR Ct scores among participants with positive results for both tests is shown in Table 3. Among the 19 participants with positive plasma qPCR results, three were positive for MTBC by blood culture. One participant with MTBC-positive blood culture had negative plasma qPCR results. Four participants had positive culture of MTBC from urine; of these, all were positive on at least one triplicate reaction. Thirty-one participants, including 29 with confirmed TB disease, had plasma tested by Xpert. Of these, 27 had a final result and two had an error on both first and second Xpert test. All 27 tests with a valid result were negative.

**Table 2:** Results of culture, Xpert and quantitative real-time PCR testing of sputum, urine, blood and plasma.

ID	Days of Treatment	HIV Status	Sputum Xpert Result	Sputum Culture Result	Plasma Xpert Result	Blood Culture Result	Urine Culture Result	Plasma DNA qPCR Replicate		
								1	2	3
1	0	+	+	+	Neg	+	+	+	ND	ND
2	0	+	+	ND	Neg	Neg	Neg	+	+	ND
3	0	+	+	ND	Neg	Neg	Neg	ND	ND	ND
4	0	+	+	ND	Error	Neg	+	+	+	ND
5	0	+	+	ND	Neg	Neg	Neg	−	−	ND
6*	0	+	+	Neg	Neg	Neg	Neg	+	+	+
7*	0	+	+	+	Neg	Neg	Neg	−	−	−
8*	0	+	+	ND	Neg	Neg	Neg	+	+	+
9*	0	+	+	+	Neg	Neg	Neg	−	−	−
10	0	Neg	+	+	ND	ND	Neg	ND	ND	ND
11*	0	Neg	+	+	Neg	Neg	Neg	−	−	+
12*	0	Neg	+	ND	Neg	Neg	Neg	+	+	−
13*	0	+	+	+	Neg	Neg	Neg	−	−	−
14*	0	+	+	+	Neg	Neg	Neg	+	−	−
15	0	Neg	+	ND	Error	Neg	Neg	ND	ND	ND
16	0	+	+	+	ND	ND	+	ND	ND	ND
17	0	+	+	ND	Neg	Neg	Neg	ND	ND	ND
18	0	+	+	ND	Neg	Neg	Neg	ND	ND	ND
19	0	+	Neg	Neg	Neg	Neg	Neg	−	−	−
20	0	+	Neg	ND	Neg	Neg	Neg	+	+	+
21	0	+	+	ND	Neg	Neg	Neg	−	−	−
22	0	+	Neg	ND	ND	Neg	Neg	−	−	−
23	0	Neg	+	ND	Neg	Neg	Neg	−	−	−
24	0	+	+	ND	Neg	Neg	Neg	−	−	−
25	0	+	+	ND	Neg	Neg	Neg	−	−	−
26	0	Neg	+	ND	ND	Neg	Neg	−	−	−
27	0	Neg	+	+	Neg	Neg	Neg	−	−	−
28	0	+	+	+	Neg	Neg	Neg	−	−	−
29	0	Neg	+	ND	Neg	Neg	Neg	−	−	−
30	0	Neg	+	ND	Neg	Neg	Neg	−	−	−
31	0	Neg	+	ND	Neg	Neg	Neg	−	−	−
32	0	Neg	+	MOTT	Neg	Neg	Neg	−	−	−
33	0	Neg	+	+	ND	ND	Neg	ND	ND	ND
34	0	+	+	+	Neg	ND	Neg	−	−	−
35	0	Neg	ND	+	Neg	ND	Neg	−	−	−
36	0	+	+	+	Neg	+	+	+	+	+
37	0	+	+	+	ND	Neg	Neg	+	+	−
38	6	+	+	+	ND	Neg	Neg	−	+	−
39	0	Neg	+	+	ND	Neg	Neg	−	−	−
40	8	+	+	+	ND	Neg	Neg	+	+	+
41	0	Neg	+	+	ND	Neg	Neg	−	−	−
42	2	Neg	Neg	+	ND	Neg	Neg	+	−	−
43	2	Neg	+	ND	ND	Neg	Neg	+	−	−
44	2	+	+	+	ND	Neg	Neg	+	+	+
45	1	+	+	+	ND	Neg	Neg	+	+	+
46	0	+	+	+	ND	Neg	Neg	−	−	−
47	0	+	+	ND	ND	Neg	+	+	+	+
48	2	+	+	+	ND	Neg	Neg	−	−	−
49	0	+	+	ND	ND	+	Neg	−	−	−
50	0	Neg	+	ND	ND	Neg	Neg	+	+	−

*3 μL plasma DNA template used for qPCR; for all other participants 2 μL used. ND = not done. MOTT = mycobacteria other than tuberculosis. Error = Xpert generated an error in two separate tests. IS6110 primers were specific to *M*. *tuberculosis* when previously tested against a panel of DNA from the following organisms: *A*. *baumannii*, *K*. *pneumoniae*, *S*. *agalactiae*, *B*. *pertussis*, *P*. *aeruginosa*, *S*. *pyogenes*, *L*. *pneumophilia*, *Pseudomonas sp*., *Sphingomonas sp*., *H*. *influenza*, *S*. *aureus*, *S*. *haemolyticus*, *N*. *meningitides*, *L*. *plantarum*, *S*. *aureus*, *S*. *intermedius*, *E*. *cecorum*, *E*. *coli*, *S*. *flexneri*, *C*. *diptheriae*, *C*. *trachomatis*, *C*. *pneumoniae*, *M*. *pneumoniae*, *S*. *pneumoniae* (M. Willby, personal communication).

**Table 3:** Cycle thresholds (Ct) of sputum Xpert and plasma real-time PCR.

No.	Sputum Xpert Ct						Plasma qPCR Ct			
	MeanCt	Probe A	Probe B	Probe C	Probe D	Probe E	Mean Ct	R1	R2	R3
1	14.2	13.2	15.3	13.4	14.6	14.4	37.8	37.8	—	—
2	15.6	14.5	15.7	15.3	16.1	16.5	38.0	37.3	38.7	*
3	15.8	15.0	16.0	15.1	16.3	16.5	36.0	36.0	*	*
4	16.0	15.3	16.3	15.8	16.1	16.6	37.1	36.6	37.2	37.4
5	17.7	16.7	18.1	17.1	18.1	18.3	42.0	—	—	42.0
6	17.8	17.2	18.3	17.6	17.7	18.2	37.9	38.3	37.6	—
7	19.7	18.7	19.9	19.4	20.2	20.5	38.4	38.6	38.3	—
8	20.7	19.4	21.3	20.5	21.3	20.9	36.5	36.1	36.7	36.6
9	22.0	20.8	22.9	21.4	22.5	22.2	38.8	38.8	38.9	—
10	22.9	22.1	23.2	22.8	23.0	23.5	39.1	—	39.1	—
11	23.1	22.4	23.3	22.9	23.3	23.8	36.5	36.1	36.4	36.9
12	23.5	22.5	23.7	23.4	23.6	24.1	39.0	39.0	—	—
13	25.2	24.3	25.6	24.5	25.8	25.7	35.0	35.4	34.6	35.1
14	26.6	25.8	26.7	26.4	26.8	27.2	33.7	33.9	33.5	33.7
15	28.7	27.6	28.7	28.5	28.9	29.6	39.7	38.1	41.3	*
16	31.0	30.5	30.3	30.0	31.5	32.5	39.0	40.7	37.9	38.4
17	33.6	32.6	32.7	32.2	35.1	35.2	38.7	38.9	38.0	39.3

Cycle thresholds for sputum Xpert and plasma real-time PCR are shown for participants with positive results for both tests. Results are ordered in ascending order of mean cycle threshold (probes A–E) for sputum Xpert. ^For plasma real-time PCR, if at least two replicates were positive, the mean of these Ct scores is shown as mean Ct; otherwise, if only one replicate was positive the Ct for that replicate is shown as mean Ct. — = No amplification; *ND = Not Done.

## Discussion

We demonstrate the ability to detect MTBC DNA by real-time PCR of plasma samples from persons with sputum smear positive tuberculosis disease, even in the absence of mycobacteremia. Our finding is consistent with the presence of circulating cell-free DNA from MTBC in the plasma of persons with TB disease. Our finding that most participants did not have MTBC-positive blood culture results suggests that bacteria in the blood were not the source of MTBC DNA detected in plasma.

Use of PCR-based methods for detection of MTBC from blood or peripheral blood mononuclear cells has been reported previously with varying yields in different patient populations^9–16^. However, blood samples were first centrifuged for preparation of whole blood cell pellets or buffy coat/peripheral blood mononuclear cells from which DNA was then isolated, a process that may be expected to concentrate whole TB bacteria as well^17^. Recently, Ushio *et al*. reported detection of MTBC DNA in plasma using digital PCR; however, culture of blood was not done and DNA was prepared from whole blood cell pellets^18^. Therefore, in the Ushio study detection of genomic DNA or DNA from whole organisms cannot be excluded. To our knowledge, the study reported here is the first to detect of MTBC from plasma in the confirmed absence of mycobacteremia.

All but one participant with positive plasma qPCR result had confirmed MTBC detected in sputum using Xpert or culture; for this one participant only sputum Xpert, but not sputum culture, was performed. Although it is not possible to rule out a false positive qPCR result in this participant, it is notable that in another participant with positive qPCR result only culture of sputum, but not Xpert testing of sputum, was positive. Further, on the qPCR reaction plate for this sample none of the other three patient samples run at the same time were positive, decreasing the likelihood of a false positive result from cross-contamination. Relative to the reference standard of culturing sputum, Xpert testing of sputum has sensitivity of approximately 90%^3^. It is therefore possible that TB disease was not confirmed due to lack of a sufficiently sensitive test.

Only two participants with positive qPCR had positive blood culture for MTBC, and none of the plasma samples tested by Xpert were positive. Xpert was initially developed for testing sputum; however, it has been successfully used to test for MTBC in a wide variety of specimen types including pleural fluid, urine and gastric aspirate and unlike culture, a positive result does not in principle depend on bacterial viability^10,19–22^. Although plasma is not a standard sample type for Xpert testing, these results further suggest that bacteria in the blood were not the source of MTBC DNA detected in plasma.

All samples that were positive by qPCR had high cycle threshold (Ct) values, and not all replicates were positive in all cases. This would suggest a low-concentration DNA target near the limit of detection, and that methods for optimization of ccfDNA recovery may be important. These results indicating low template concentration are similar to a recent report of another novel sample type, oral mucosal swabs, for TB diagnosis by real-time PCR testing^23^. Yield of ccfDNA has been shown to vary according to collection and processing procedures^21,24^. Detection of target DNA may be improved by specialized blood collection tubes that minimize release of human DNA from the lysis of white blood cells^25,26^. Sputum Xpert MTB/RIF Ct scores did not show a clear correlation with plasma qPCR Ct values, suggesting there is not a straightforward association between sputum bacterial load as assessed by Xpert MTB/RIF Ct score and detection of plasma DNA. Alternatively, differences in specimen processing (e.g. time from collection to processing) could potentially lead to differences in yield of purified DNA and qPCR sensitivity.

Based on our results, plasma DNA may be a useful target for TB diagnostic testing. Given the modest sensitivity of qPCR compared to Xpert, along with the delayed Ct values observed, improvements in the sensitivity of plasma DNA recovery and detection would be important. This is particularly true as the target for plasma qPCR in this study, IS6110, is a repetitive genetic element in *M*. *tuberculosis*, and therefore sensitivity of qPCR for this target may be higher than for other common targets for TB detection including those for detection of drug resistance, such as *rpoB*. The qPCR assay tested here also generates a very short amplicon which, depending on the size of target DNA in plasma, could enhance its sensitivity compared to alternate qPCR assays dependent on longer amplicons.

Improvements in the sensitivity of plasma TB detection would be important for its potential utility as a diagnostic test. Based on studies of human ccfDNA, methods of optimizing target DNA recovery may be expected to improve molecular detection of TB DNA in plasma. Improvements in the detection rate of ccfDNA in oncologic applications using developing technologies gives reason to be optimistic that improvements in the sensitivity of detection of plasma tuberculosis DNA may be feasible^6^. This proof-of-principle study included only persons with tuberculosis disease readily diagnosed by sputum smear. Positive sputum smear result is generally indicative of high burden of disease in the lung, but tuberculosis can affect many organ systems and also disseminate widely in the body without resulting in sputum smear positivity. Further study is needed to better determine characteristics of persons with TB disease in whom ccfTB DNA might be detected in plasma. If detected over a broad range of clinical manifestations and severity of disease, TB plasma DNA could provide a target for improving clinicians’ ability to diagnose TB in patients in whom collection or testing of sputum has poor diagnostic yield (e.g., children, people living with HIV, people with extrapulmonary TB). In addition, because we demonstrate detection of TB DNA in plasma, these findings suggest that DNA-based applications such as molecular drug resistance testing may be feasible from plasma. If true, this would represent a significant advantage over methods that rely on sputum or on slow mycobacterial culture.
